# Use and value of systematic reviews in English local authority public health: a qualitative study

**DOI:** 10.1186/s12889-020-09223-1

**Published:** 2020-07-13

**Authors:** Emily South, Theo Lorenc

**Affiliations:** Centre for Reviews and Dissemination, University of York, York, YO10 5DD UK

**Keywords:** Systematic reviews, Evidence, Policy-making, Local government

## Abstract

**Background:**

Responsibility for public health in England transferred from the National Health Service to local authorities in 2013, representing a different decision-making environment. Systematic reviews are considered the gold standard of evidence for clinical decision-making but little is known about their use in local government public health. This study aimed to explore the extent to which public health decision-makers in local authorities engage with systematic reviews and how they do so.

**Methods:**

Semi-structured interviews were conducted with senior public health practitioners (*n* = 14) in Yorkshire and the Humber local authorities. Sampling was purposive and involved contacting Directors of Public Health directly and snowballing through key contacts. Face-to-face or telephone interviews were digitally recorded, transcribed verbatim and analysed using the Framework Method.

**Results:**

Public health practitioners described using systematic reviews directly in decision-making and engaging with them more widely in a range of ways, often through a personal commitment to professional development. They saw themselves as having a role to advocate for the use of rigorous evidence, including systematic reviews, in the wider local authority. Systematic reviews were highly valued in principle and public health practitioners had relevant skills to find and appraise them. However, the extent of use varied by individual and local authority and was limited by the complexity of decision-making and various barriers. Barriers included that there were a limited number of systematic reviews available on certain public health topics, such as the wider determinants of health, and that the narrow focus of reviews was not reflective of complex public health decisions facing local authorities. Reviews were used alongside a range of other evidence types, including grey literature. The source of evidence was often considered an indicator of quality, with specific organisations, such as Public Health England, NICE and Cochrane, particularly trusted.

**Conclusions:**

Research use varies and should be considered within the specific decision-making and political context. There is a need for systematic reviews to be more reflective of the decisions facing local authority public health teams.

## Background

In 2013 responsibility for public health decision-making in England transferred from National Health Service (NHS) primary care trusts to upper-tier and unitary local authorities (LAs) as part of the Health and Social Care Act 2012. LAs became responsible for commissioning a number of specific public health functions, such as sexual health services, smoking cessation, drugs and alcohol services and obesity programmes, with a ring-fenced budget provided to deliver these functions [1]. As well as commissioning these services, it was intended that public health teams could influence decisions on policy and commissioning within other parts of the authority that impacted the wider determinants of health, such as housing [1]. Each LA has a Director of Public Health (DPH), which is a statutory position held by a consultant in public health, responsible for leading on public health locally [2]. DsPH are supported by other consultants, who are qualified as public health specialists and registered with a professional body [2].

Public health has long been viewed as an evidence-based discipline with principles based on evidence-based medicine (despite critiques of this framing) [3]. Senior public health professionals are trained in evidence use as part of the five-year training programme (or equivalent experience) prior to becoming registered consultants [4]. However, LAs represent a different policy-making environment to the NHS in a number of ways. Firstly, elected politicians are involved in decision-making [5–7], which has the potential to impact on evidence use [6]. Secondly, research has shown that cultures of evidence use are different in non-health sectors, such as transport, planning and housing, with which public health teams are now expected to work [8]. There have also been large reductions in the ring-fenced public health grant since the transfer [9] and there is evidence that public health practitioners (PHPs) see themselves as having less influence or status in some ways since the transfer [5, 7, 10].

Systematic reviews are an important type of evidence for public health practice as they synthesise all available primary research studies to provide a more reliable estimate of intervention effectiveness [11], or a reliable overview of findings on issues such as disease prevalence and risk factors for developing a disease [12]. While, historically, much of the initial development of systematic review methodology took place within medical research, its application to public health questions has proceeded rapidly, and is now widely accepted as an important part of the evidence landscape informing public health policy [13, 14]. This expansion of domain has in turn facilitated a shift in methods, as systematic reviewers have realised the limitations of a model focused mainly on reviewing randomised trials. Increasingly, systematic reviews are investigating barriers and facilitators to implementing interventions and exploring the views and experiences of different stakeholders [12]. There is also a range of other types of evidence review available (e.g. scoping reviews, rapid reviews), generally characterised by less rigorous methodology [15]. Systematic reviews are considered the gold standard of evidence-based decision-making in clinical settings [16] but there have been criticisms of the systematic review evidence base in public health. These include a reliance on rigorous study designs that are less widely-used in public health research [17], few reviews on the social determinants of health [18], a large number of reviews with uncertain conclusions [19], and a lack of consideration of policy implications [20].

Given the number of systematic reviews published and the growing literature on strategies to improve their uptake in decision-making [21–23], it is important to understand how practitioners in different contexts perceive their value and use. Although some studies have explored systematic review use in public health [24, 25], much of the evidence use literature considers academic research in general [6, 8, 26]. A more in-depth understanding of the barriers and facilitators to systematic review use in LAs could help systematic review authors and commissioners to better meet the evidence needs of PHPs. Generally research has found relatively limited direct use of academic evidence in policy-making [6, 8, 26]. However, potential complexities in the relationship between evidence and policy have been highlighted, for example the argument that research should focus on the influence of ideas on policy [27].

There has been limited research on the use of evidence in LAs since the transfer of public health. A systematic scoping review published in 2017 identified eight studies on local public health decision-making in England that explored evidence use after the transfer to some extent [6], although a number have been published since then [10, 28–32]. To our knowledge, there has been no study focusing specifically on the use of systematic reviews in the context of local public health decision-making since the transfer. The aim of this study was to explore the extent to which public health decision-makers in LAs engage with systematic reviews or other evidence reviews and how they do so [33]. Given the complexities in the evidence-policy relationship, this was explored in terms of both direct use in decision-making and wider engagement. Decision-makers were defined as PHPs working within LA public health teams and contributing to the decision-making process for policy or commissioning decisions in public health or other related areas.

## Methods

Semi-structured qualitative interviews were conducted with PHPs in the Yorkshire and the Humber region.

### Sample

Sampling was purposive and targeted senior PHPs in LAs, specifically DsPH and public health consultants, as they were considered most likely to be able to provide insight into decision-making. Recruitment was undertaken in two phases. Firstly, the DsPH at all 15 upper-tier and unitary LAs in the Yorkshire and the Humber region were invited to participate in the study or provide details of a colleague who may be able to take part. The second phase involved snowballing through key contacts to recruit further participants. Although sampling focused on DsPH and consultants, other public health staff were interviewed where specific individuals were recommended as having useful insight into the use of evidence by the DsPH contacted or through snowballing. All potential participants were invited to participate through email with an information sheet provided. Sampling was iterative and continued until data saturation was judged to have been reached by the researcher conducting interviews.

### Procedure

All participants provided written informed consent. An interview schedule was developed for the study, with questions derived from the study aims but tailored to different job roles (see Additional file 1). Participants were asked about their role and involvement in making or supporting decisions, the direct use of reviews in policy-making or commissioning (including identifying reviews and any assessment of quality), other ways in which they engaged with research evidence and the value and use of reviews in relation to primary research. They were also asked to reflect on how valuable systematic review evidence was, anything that could be done to improve the usefulness of reviews and how important it was that review methods were robust. Interviews were conducted face-to-face or over the telephone by one researcher (ES). Interviews were recorded using a digital recorder and transcribed verbatim, with identifying information removed. Ethical approval was granted by the University of York Health Sciences Research Governance Committee**.**

### Analysis

Interview data were analysed using the Framework Method [34], as it allows analysis of themes across interviews, while retaining a sense of the views of each individual participant [35]. In this case it was important to consider data within the context of the role of each participant (e.g. DPH, consultant). The Framework Method is appropriate in studies where all interviews covered similar issues, and it can be used with an inductive, deductive or combined approach [35]. In this case a combined approach to analysis was used, with a framework that included deductive themes based on in the interview schedule and inductive themes identified through open coding of five interviews. NVivo 12 was used to code transcripts with the framework themes and generate framework matrices for each of the seven main categories. Coded data was summarised into the matrices. The framework was revised as necessary throughout coding and analysis. Coding and analysis were conducted by one researcher (ES), with framework matrices and the final analysis checked and refined by a second (TL).

## Results

### Participants

Fourteen interviews were conducted between June and September 2018, with participants from ten of the 15 LAs in the region. One LA declined to participate due to time constraints and four DsPH did not respond. Five participants were DsPH, five were consultants and one was a public health speciality registrar. The remaining three were other PHPs, all of whom had a part of their remit related to evidence or research use. Eight participants worked for metropolitan district councils (covering urban areas) and six worked for non-metropolitan councils. These LAs represented a mix of deprived and more affluent areas, according to the English Index of Multiple Deprivation [36]. Interviews lasted between 19 and 46 min. Two were conducted over the telephone and the remainder were face-to-face at LA offices.

Findings have been grouped below under the thematic categories used in the framework analysis and illustrated with anonymous quotations from the data. The first four categories focus on the utilisation of systematic reviews in decision-making. The remaining categories describe themes that emerged around the broader context of evidence use and the role of PHPs within the local authority. Key findings under each thematic category are summarised in Table 1.

**Table 1:** Summary of key findings from framework analysis

Thematic category	Key findings
Use of systematic reviews in decision-making	• Systematic reviews used to some extent in commissioning and policy-making. • Other evidence used alongside or instead of reviews, with grey literature from specific organisations highly valued. • Use of systematic reviews varies between individuals, job roles and LAs.
Finding and selecting reviews	• PHPs have search and appraisal skills but time is a barrier. • Literature often found through quick searches or other routes and quality assessed informally. • Diverging views on the value of non-systematic reviews, with factors other than methodology more important for some PHPs.
Barriers to use	• Limited number of reviews on some key topics in LA public health, narrow focus of reviews and restricted access to full texts are all key barriers. • Other barriers include generalisability to place and LA context and the involvement of politicians in decision-making.
Improving the usefulness of systematic reviews	• Suggestions included providing executive summaries, recommendations for practice and economic evidence.
Role of PHPs in advocating for evidence use	• Senior PHPs perceive themselves as having role advocating for use of evidence in wider LA. • Commitment to evidence use linked to professional identity. • Evidence used in range of ways to influence or persuade, including symbolic use and more nuanced ways.
Engagement with research outside decision-making	• PHPs frequently engage with research, often through a personal commitment to maintaining knowledge and professional development.
Value of systematic reviews in LA context	• Systematic reviews highly valued in principle but limits to value and impact in LAs. • Decision-making is complex and there are contesting interpretations of evidence, with local and anecdotal evidence valued.

LAs Local authorities; PHPs Public health practitioners

### Utilisation of reviews

#### Use of systematic reviews in decision-making

Participants considered use of the evidence base to be an important part of public health decision-making:

“it’s certainly part of the culture of our public health team and I would say all of our services, when we are looking at reviewing them, when we’re looking at service specifications etc., one of the things we do is what’s the best available evidence and … that’s something that we keep looking for” (DPH02).

As part of this, systematic reviews were used by all public health teams to some extent; both in directly informing public health decisions and influencing decisions in the wider LA. Examples of systematic review use mainly involved the commissioning of services, including use in needs assessments and service specifications, but there were also examples of use in policy-making. Examples ranged from completely proactive use at the start of the decision-making process to reactive use, supporting or refuting a proposed decision.

However, participants did not rely exclusively on systematic reviews and discussed using other types of evidence alongside or instead of them, including grey literature and primary research. In particular, evidence summaries or briefings from trusted organisations and National Institute for Health and Care Excellence (NICE) guidance were highly valued and to some participants comparable to systematic reviews. Most participants mentioned specific trusted organisations, particularly Public Health England (PHE), NICE and the King’s Fund, with one consultant explaining that NICE and PHE were seen as “unbiased” and “independent” (Consultant05). It was pointed out that these summaries often collate evidence from reviews or are “reports … which … blur the gap between a systematic review and other things” (DPH01). When asked about systematic reviews, participants would often talk about these grey literature reports instead and some used these more often than systematic reviews. For example, one DPH said that their team tended to use evidence through “briefings rather than actually going to search for the actual systematic review” (DPH05). The approach to using primary research ranged from some participants who highlighted the risk of basing decisions on single studies to others who believed the relative value of reviews and primary research depended on factors such as the decision, the audience or the context the primary research was undertaken in:

“I think they're both equally important...it does just depend on what's available and … the question you're trying to answer. I wouldn't put one higher than the other” (Consultant02)

The extent of systematic review use varied by remit, personal approach to evidence and role, with consultants and other PHPs using them more than DsPH. One of several consultants who used them extensively explained how this may not be widespread:

“I've got colleagues who work on other parts of public health … they aren't regularly … accessing journal articles so I'm kind of like well … how's evidence driving what you do? And I think they're probably more reliant on a lot of the policy stuff that comes out of Public Health England for example” (Consultant03)

Systematic review use also differed between LAs. While it was clearly part of the culture in some public health teams to regularly consider research evidence in decisions, a few participants suggested that their team had less of a systematic approach to incorporating evidence:

“We're moving in that direction. I don't think as a local authority we particularly have a history of doing that” (Other01)

#### Finding and selecting reviews

Participants had literature searching and critical appraisal skills but in practice these were not always used, with time a significant barrier:

“it’s really difficult the way you work here, you don't have the time to kind of do the kind of critical appraisal type approach that you would in other settings possibly” (Consultant03)

Some participants stressed that literature searches were quick and most participants also found systematic reviews through other routes, including through colleagues and networks, Google and trusted online sources. Quality was often assessed informally rather than through critical appraisal tools. There was a theme of relying on “gut instinct” (Consultant03) and making intuitive, quick judgements on quality or using reviews from sources that were trusted to be of good quality. Trusted sources of systematic reviews that were mentioned included Cochrane and specific academic departments in the UK.

There was a range of views on the value of non-systematic evidence reviews (e.g. rapid reviews). Some participants expressed a preference for systematic reviews, but acknowledged that sometimes there was a need to use other types of review, for example in areas with limited evidence. For others, it depended on the situation or other factors that were considered more important than methodology, such as reliability, accessibility, source or face validity:

“I think some of the distinction between working in practice public health and working in academic public health is that in academic public health we get really caught up with methodology and labelling and the purpose of this and … what it is and what it isn't and I think in practice public health, if the publication has face validity in that feels like it hangs together and it resonates with your experience and it feels practical so it’s got some actionable things in it, then even if you had concerns about the robustness of the methodology then you'd probably still think it was worth thinking about” (Other03)

#### Barriers to use

Barriers to using systematic reviews within LAs included availability of relevant reviews, the narrow focus of research questions, access to journal articles, and the involvement of politicians who may favour other evidence types in decision-making.

The limited number of relevant systematic reviews available was viewed as a key barrier. Participants discussed how there was much more systematic review evidence available for healthcare public health and other clinically-focused topics, compared to the wider determinants of health, communities and social care:

“the local authority is a social model of public health whereas when we were in the NHS, it was more of a clinical model of … public health, although we did do the social aspects as well but this is much more around the social determinants of health, the causes of the causes. So actually there might be opportunity to do systematic reviews moving forward that really do focus … on our new function, role and opportunities” (DPH05)

Most participants had to use primary research at times because of limited availability of systematic reviews:

“Often I think with the areas I'm interested in, like wider determinants, adult social care, there it would always be primary research because they just there isn't a lot of other stuff.” (DPH03)

However, there were opposing views, including one consultant who said that there were “loads of good social care papers and systematic reviews out there” (Consultant04). Systematic reviews were also seen as being narrow in their focus by some participants, as they tended to cover single issues, while public health decisions could be complex:

“systematic reviews still tend to be quite topic-based. So you'll get a systematic review on smoking cessation or a systematic review on physical activity or one on alcohol consumption. You won't or less common would be to find a systematic review that talked to you about community development and creating safer environments for people to live in.” (Consultant01)

A few participants raised the limited number of systematic reviews on how to organise services, highlighting that this was the subject of many public health decisions. It was also suggested that more reviews that addressed context through qualitative or realist methods would be useful for decision-making.

A significant barrier was restrictions on access to full journal articles, which was a major source of frustration for some participants:

“I would say there's a huge problem with accessing publications. So if you do find stuff that you want to look at the original article, I mean … I've got an Athens password so that's great, I can get into what the NHS has subscribed to but that is so limited” (Consultant02)

While some public health teams worked around the limited access, others mentioned using abstracts only or relying on open access publications.

Most participants highlighted that systematic reviews were not always applicable to the context they were working in, in terms of generalisability to population and place or taking into account the LA context. Primary research undertaken in a local or similar context was particularly valued:

“I think you need to start with the systematic review, that gives you the overall picture, but very often you would find that it's the localised studies that then are the ones that are probably more powerful in terms of moving things” (DPH02)

Issues were raised around whether systematic review findings could be translated and implemented within the specific context of LA decision-making, taking into consideration local issues such as budget and remit.

Some participants felt that evidence was viewed differently in a political organisation, with anecdotal evidence from constituents and local evidence particularly valued by councillors. Some participants also saw political ideology or strategy as a barrier to implementing evidence:

“you can have all the evidence, gold standard evidence that says this is the course of action but there may be competing evidence from a political ideology perspective or whatever that y'know just means sorry but we ain’t going to do it” (Consultant02)

However, again, there was some divergence on this:

“we usually have … robust conversations but we don't have to sort of compromise really in what … we want to do. The council and politicians generally do accept the evidence-based recommendations.” (Consultant05)

#### Improving the usefulness of systematic reviews

Other than addressing the barriers above, participants were able to suggest several specific ways that systematic reviews could be improved to be more useful. It was suggested that good executive summaries were important, particularly due to time constraints. Several participants also spoke of the value of systematic reviews providing recommendations on implementation and practice. The inclusion of economic evidence, such as return on investment, in systematic reviews was also suggested:

“if it's a systematic review that also includes … cost effectiveness evidence so including the economic basis, that often can help broaden the arguments so it moves from just being does this intervention work … [to] actually is there a good financial reason for doing it?” (Registrar01)

Participants from two LAs wanted better links with universities, in terms of input into research priorities, academics visiting LAs to explain their research or translation of systematic reviews into guidance for practice:

“I suppose that's part of what we try to do locally is translate the systematic reviews into useful pieces but sometimes we don't have the connection with the academics that we could benefit from.” (DPH01)

One DPH was interested in the potential for more secondments of academics to LAs and vice versa. Another issue raised was the need for systematic reviews to be disseminated so that PHPs were aware of new research. An online resource where all systematic reviews could be quickly found and accessed and email updates on newly published reviews were both suggested.

### Evidence and professional roles in the LA context

#### Role of PHPs in advocating for evidence use

A key theme that emerged was that senior PHPs saw themselves as having a role advocating for the use of evidence within LA decisions.

For some, this commitment to evidence use was clearly linked to their sense of identity as a public health professional. For example, a number of consultants and DsPH referred to their public health training or were self-reflective about their use of evidence. Some expressed feelings of regret or sadness that they no longer employed skills such as literature searching or critical appraisal, or kept up-to-date with research:

“I feel like I've let that side of my discipline go away because I'm so busy just trying to do the day job … and some Directors of Public Health I know are much better at keeping up with evidence or … good at having like … an area of interest that they keep up to. I think I rely a lot on experts … or when I do go to kind of CPD events but … it's a failing of mine really that I should spend a bit more time keeping up with it” (DPH03)

PHPs were involved in trying to influence decisions beyond the public health team, including those of elected members and other parts of the LA, such as social care and the wider determinants of health. Some participants also saw themselves as having a role to interpret and summarise evidence for decision-makers:

“I see myself as a bit of a broker between kind of taking the academic information and presenting that in a way that's meaningful to kind of the audience that I'm working with, be it kind of planners or commissioners or clinical leads.” (Consultant03)

As part of this influencing role, some teams used specific pieces of evidence to persuade and influence decision-makers to support a certain position:

“there's the more reactive stuff where something's happening or somebody says something and...I then have to go and look for evidence to either support or refute or we know that there's evidence that supports or refutes but having to sort of put it forward to change the direction of travel I suppose” (Consultant02)

Some of this was akin to what has been described as political or symbolic evidence use, where evidence is used to justify a pre-determined position [37, 38]:

“Sometimes it can be held up as 'well this isn't just my idea, these people are saying this' and that can be quite helpful” (Other03)

However, evidence was also used in more nuanced ways:

“… we live in a system that’s intrinsically unfair and I do think there’s something around evidence challenging some of those power structures that are really helpful” (DPH03).

“they [systematic reviews] can also help with changing thinking about something … they may help us think through a particular issue or they may help us commission a bit of research locally or they may help us have conversations with people. So I think they have a role but … I don’t think it’s that- and I don’t think to be fair I don’t think it’s ever been that really linear process” (Other03).

Another example of evidence use that was distinct to direct instrumental application to existing problems was the active response to new evidence as it was published:

“I suppose … the other thing that we might look at, the other way that we'd use evidence, whether systematic reviews or individual studies, would be what we do when they come out. So there might be significant ones published and then there’d be the question of we would then potentially look at our policy and how we might want to develop our policy based on those.” (DPH04)

#### Engagement with research outside decision-making

PHPs frequently engaged with systematic reviews and other research outside decision-making processes. The main reasons for this were maintaining knowledge, professional development and interest and it was sometimes expressed as a personal commitment. For example, one consultant had personal subscriptions to journals. Another consultant described themselves as a “big evidence person”, explaining that:

“I understand healthcare and social care through the research. That's just the way I've always done it.” (Consultant04)

However, two consultants regarded it as an integral part of their job to keep up-to-date with new evidence:

“we use evidence and systematic reviews and published evidence summaries from NICE and other national organisations … just as part of our day job really. We're always on the lookout for new evidence, new summaries, any changes … that might shift our knowledge and our thinking” (Consultant05)

Research was encountered through a wide range of different routes, including dissemination from other organisations, Twitter, conferences, journal clubs, and professional networks.

#### Value of systematic reviews in local authority context

Despite varying use, systematic reviews were highly valued in principle by most participants, particularly consultants, and seen as an important contribution to public health work:

“they definitely have a place, they're definitely very useful and we need to have them, even if they're not always acted on” (Consultant02)

Their value was expressed by some participants as their ability to save them time by preventing the need to search for, appraise or summarise literature themselves. Relatively few participants explicitly stated that they valued them because they were high quality evidence but their perceived quality was often implicit when participants discussed their use. Some participants described a feeling of comfort or lack of worry when using systematic review evidence:

“the sort of comfort of knowing … there’s somebody who’s done this really, really well” (Consultant04).

However, some participants expressed limits to the value and impact of systematic reviews within LA decision-making. DsPH tended to talk more about the limits of reviews and the complex nature of decision-making. A theme emerged that published evidence was “only part of the puzzle” (DPH02) in decision-making:

“it's only the start of the process to … build your case. The important thing is being able to argue your case and influence from that. So … for example you can design your service, you can start your policy on the basis of the evidence but you can't [be] quoting evidence to people indefinitely because it doesn't you don't come across well. You have to be able to put … everything into context and also have to reflect reality of funding, … political decisions” (Registrar01)

There were also contesting interpretations of evidence within LAs and a limited understanding of systematic reviews and other research outside of public health teams was highlighted. One consultant described how this could lead to difficult conversations when non-public health colleagues presented evidence that may be of limited quality or relevance. As previously discussed, a key theme that emerged was the power of local and anecdotal evidence within LAs:

“I think there's a broader notion of what constitutes evidence and not quite so much the hierarchy of evidence that I'm used to from a more NHS, medical … gold standard systematic reviews through to observational stuff or whatever. Whereas … there's a hierarchy in that setting and I think in local government it's more horizontal so what a handful of people have said in a focus group is considered to be as important as a systematic review” (Consultant02)

Trying to introduce research evidence to counter this was not always effective and could be received badly:

“Sometimes the word ‘evidence’ or ‘this has come from the research’ doesn't always go down well with members and some of that is because local government really likes stuff that's relevant locally.” (Other03)

In particular, international evidence and research from other parts of the UK could be disregarded or challenged by elected members:

“whereas doctors, whereas health people I think will hold national evidence quite strongly, I think with when people work very locally, they're a bit like 'well that's all well and good but it doesn't apply to me' a bit more” (DPH03)

Despite the contesting interpretations of evidence, the promotion of evidence within the LA was not always framed as PHPs working against an environment that was resistant to academic evidence. One participant said that, within a stakeholder group, public health would be seen as “the evidence guru” (Registrar01) and relied on to interpret the evidence base. Some other participants suggested that public health teams had the potential to increase skills and understanding of evidence within the wider LA.

## Discussion

Interviews with PHPs demonstrated that systematic reviews are used directly in decision-making, as part of a culture of evidence use in the LA public health workforce. A wider engagement with systematic reviews and other research that is arguably intrinsic to senior public health roles, and sometimes expressed as a personal commitment to developing knowledge, was also noted. PHPs saw themselves as having a role advocating for the use of evidence within the LA and in some cases translating the academic evidence base. However, the extent of systematic review use varied between individuals and LAs. Use can be limited in the LA context, despite the fact systematic reviews are highly valued in principle and PHPs have a high level of exposure to research and appropriate skills to use it. Evidence is seen as only one factor in decision-making processes that can be complex and there are contesting interpretations of evidence. There are also a number of barriers to systematic review use in LAs, including time constraints, involvement of politicians and restricted access to journal articles. There are also barriers associated with the research available, including a lack of reviews published on topics relevant to LA public health. Systematic reviews can be seen as too narrow in their focus and not sufficiently reflective of the complex decisions facing PHPs. Other evidence is used alongside or instead of systematic reviews, with grey literature reports from specific trusted sources particularly valued. The source of evidence was important to participants and used as a quick way to judge quality and reliability.

This study confirms findings from previous studies on public health decision-making which found that a wide range of evidence beyond academic research is used, local knowledge and evidence is particularly valued, and anecdotal evidence is powerful [3, 6, 8, 26, 28, 39, 40]. Credibility of evidence is often determined through the reputation of the author or institution rather than the methodological quality of the research [8, 25]. Findings confirmed a number of practical barriers identified in previous studies, including access to research [6, 8, 26, 28, 41]. Findings are also consistent with recent research on LAs that has highlighted the role of PHPs in framing evidence for others and the impact of politics on evidence use [28, 40]. However, compared with other evidence types, research use has been seen as limited and mainly symbolic or conceptual (influencing decisions indirectly by contributing to general enlightenment [37, 38]) rather than instrumental (directly applied to a problem [37, 38]) [8, 26, 39]. Specifically, while previous research has found evidence in support of systematic review use in public heath settings [25, 26, 39], there was limited instrumental use in decision-making [25] and reviews were perceived as less valuable than some other evidence types [25, 39]. While this study reiterates some of the limitations of systematic reviews, it found that they were used extensively and often instrumentally by some, albeit not all, PHPs, who valued them highly. For some participants, the use and promotion of rigorous evidence, including systematic reviews, was an important part of their identity and role as a public health professional.

However, this study raises questions over how easily examples of evidence use can be categorised as instrumental, symbolic or conceptual in political contexts. Although participants described research evidence being used in a ‘tactical’ manner, this did always equate to symbolic evidence use. In this context, even policies or positions that had been informed instrumentally by evidence still had to be sold politically to elected members, sometimes requiring the tactical use of specific pieces of evidence. A few participants also spoke about implementing new research findings as they were published, which is closer to the knowledge-driven model described by Weiss [37].

Findings reiterate the complexity and heterogeneity of decision-making processes and evidence use [6, 26]. They also reinforce earlier findings that the idea of ‘evidence-based’ policy does not adequately capture the complexity of evidence-policy relationships in practice [3, 27, 42]. Conventional hierarchies of evidence may not address the broad, systemic nature of the challenges faced in public health practice (as opposed to narrowly defined research questions), or other stakeholders’ divergent preferences for evidence. Use of systematic reviews can be limited by a perceived lack of relevance and systematic review authors and commissioners should consider widening the evidence base to meet public health needs, as has previously been suggested [28]. The systematic review community has begun to recognise the need to address questions of relevance to practice and policy, and is now more routinely involving stakeholders in question-setting [43] and developing methods to address more complex questions [44]. There are also more efforts to take account of context [45, 46] and implementation [47] issues in systematic reviews. Recent discussion has also focused on how evidence syntheses such as systematic reviews can better meet the needs of policy-makers across all policy areas, including involvement of policy-makers throughout, accessible language and open access publication [48]. Lack of time and access to publications are barriers that would need to be addressed within the public health community, although both may prove difficult to address without increased public health funding. The positive framing of the promotion of evidence use by some PHPs suggests that there may be opportunities for public health to play a role in increasing appetite and capacity for using research across the wider LA.

### Limitations

This study was based on a limited number of interviews in ten LAs. It was clear that many of the participants had a particular interest in evidence use so the findings may not reflect the full range of attitudes towards systematic reviews held by PHPs. Sampling focused on senior PHPs who had completed the public health speciality training programme and findings may not be generalisable to staff who have received less training in evidence use. As participants were working in one region of England only, some findings may not be generalisable to LAs in other regions. It has been suggested that there are specific drivers of health inequalities in the North of England and thus different priorities to other regions [49]. As highlighted by the findings of this research, context is important and not all findings will apply to other public health settings. Participants were aware that the interviewer worked for a university department specialising in systematic reviews, which may have influenced some of their responses. This also introduces possible bias into data collection and analysis. The authors may have shown bias towards more favourable views of systematic reviews, given their own views, although authors were conscious of this risk and effort was taken to avoid this.

## Conclusions

This study contributes to the understanding of the use of systematic reviews in decision-making, and more specifically the use of evidence in LA public health after the transfer of decision-making from the NHS. While it confirms a number of barriers and limitations to systematic review use found in previous studies, it also found differences with research undertaken in other public health settings. For example, some individuals used systematic reviews extensively, including direct use in decision-making processes and wider engagement, albeit alongside a range of other evidence. This reiterates the importance of considering evidence use with reference to the specific context and actors involved, in this case senior PHPs in LAs. Findings also highlight the complexity of decision-making and the fact that systematic reviews need to be more reflective of the decisions facing PHPs in LA public health.

## Supplementary information

**Additional file 1.** Interview schedule.

## Data Availability

The dataset generated and analysed during the current study is not publicly available and cannot be shared due to confidentiality, as participants are potentially identifiable from the information contained in the data.

## Abbreviations

DPH: Director of Public Health
LA: Local authority
NHS: National Health Service
NICE: National Institute for Health and Care Excellence
PHP: Public health practitioner
