# Supplementary material for: Use and value of systematic reviews in English local authority public health: a qualitative study
Source: BMC Public Health. 2020 Jul 13;20:1100. doi: 10.1186/s12889-020-09223-1 (PMC7359488; doi:10.1186/s12889-020-09223-1)
Supplement: Supplementary file 1 — Additional file 1. Interview schedule. [file 12889_2020_9223_MOESM1_ESM.docx]

**Interview schedule**

*Please note that small changes were made to the schedule for some interviews to tailor it to the job role of participants.*

**Questions about role**

Please can you briefly explain your role and how you’re involved in making (or supporting) decisions on policy and commissioning?

**Direct role of reviews in policy-making**

When you make (or support) policy or commissioning decisions do you ever use published evidence reviews, such as systematic reviews, in this process? If so, how?

*Prompts:*

- Examples?
- Reasons for using/ not using reviews? (if not, ask what using instead)
- At what stage of the process?
- How exactly use within decision-making process. What role?
- Policy/commissioning

How do you identify which reviews to use?

*Prompts:*

- Accessibility
- Source
- Timeliness
- Quality- critical appraisal

To what extent do you think reviews are useful in the decision-making process?

*Prompts:*

- Why/why not?
- Some topics more than others?
- Local authority context
- Examples?
- Barriers?

**Other ways of engaging with reviews**

Excluding this direct use in decision-making, are there other ways you use, read or engage with published evidence reviews in your day-to-day work?

*Prompts:*

- Specific ways do this?
- Do they have an important role?
- Recent example?

Are you able to give me an example of a specific review that has been really helpful to your work? (decision-making or general use)

Does it matter to you whether a review is systematic or uses less robust methods when using it in your work?

**Value and use of reviews in relation to primary research evidence**

In what circumstances would you use primary research studies over a review?

*Prompts:*

- Examples?
- What kinds of primary research are useful?
- Which are more useful or relevant for your work in practice? Why?
- What are the differences, if any, in how you use reviews and primary research studies?

Just to sum up, how valuable would you say systematic review evidence is in the overall picture of the work you do?

Is there anything that could be done to improve their usefulness for decision-making in LAs?

Is there anything more you want to add?
